# Supplementary material for: Carbon Nanotubes and Polydopamine Modified Poly(dimethylsiloxane) Sponges for Efficient Oil–Water Separation
Source: Materials (Basel). 2021 May 7;14(9):2431. doi: 10.3390/ma14092431 (PMC8125137; doi:10.3390/ma14092431)
Supplement: Supplementary file 1 [file materials-14-02431-s001.zip › materials-1209824-SM-final.pdf]

materials

Supplementary Materials

Carbon Nanotubes and Polydopamine Modified Poly(dimethylsiloxane) Sponges for Efficient Oil–Water Separation

Wen Zhang [1], Juanjuan Wang [2,*], Xue Han [2], Lele Li [1], Enping Liu [1] and Conghua Lu [1,2,*]

[1] School of Materials Science and Engineering, Tianjin University, Tianjin 300072, China; zhangjike445@tju.edu.cn (W.Z.); lelelee86@163.com (L.L.); epliu@tju.edu.cn (E.L.)
[2] School of Materials Science and Engineering, Tianjin Key Laboratory of Building Green Functional Materials, Tianjin Chengjian University, Tianjin 300384, China; hanxue@tcu.edu.cn
* Correspondence: wangjj@tcu.edu.cn (J.W.); chlu@tju.edu.cn (C.L.)

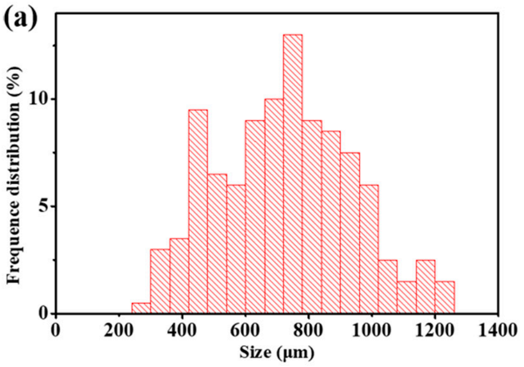

Figure S1. The particle size distribution diagram of r-CAM (**a**) and g-CAM (**b**).

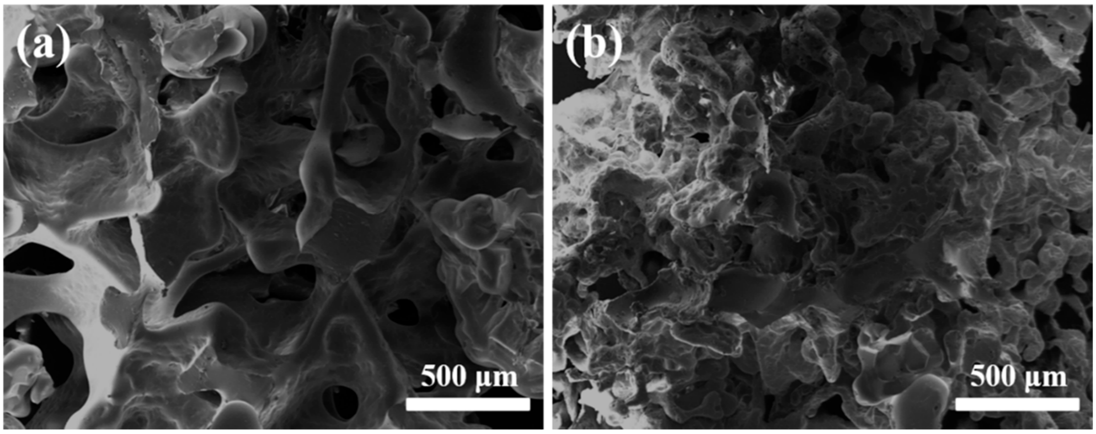

Figure S2. SEM images of porous PDMS prepared with r-CAM (**a**) and g-CAM (**b**) template.

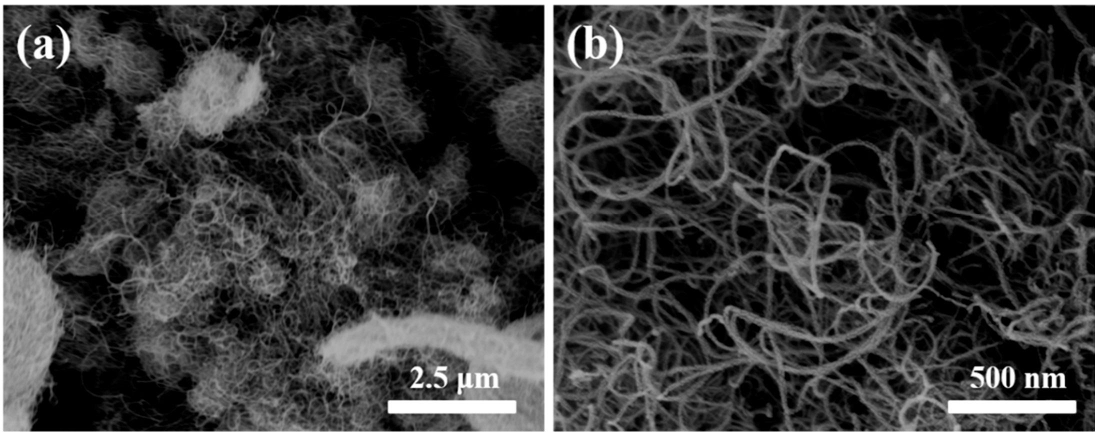

Figure S3. SEM images of CNTs used to modified porous PDMS.

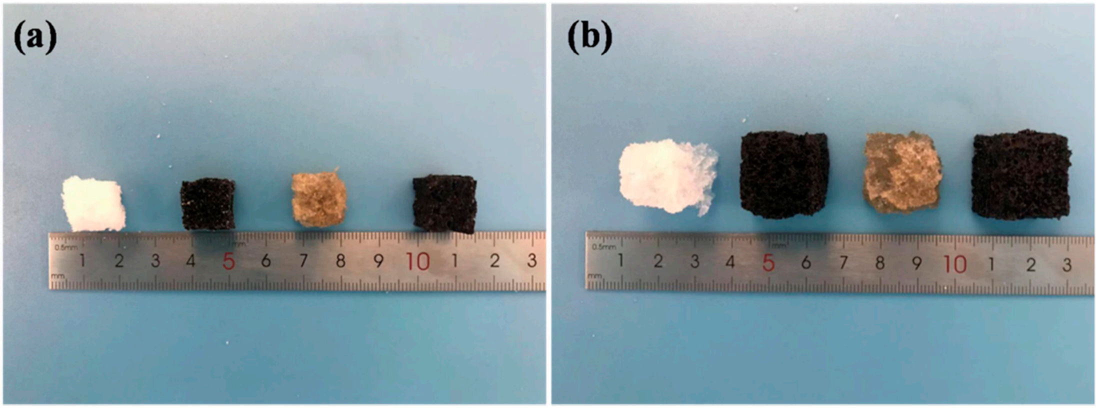

Figure S4. The volume change of PDMS, CNT-PDMS, PDA/PDMS and PDA/CNT-PDMS (from left to right) before (**a**) and after (**b**) soaked in cyclohexane solution.

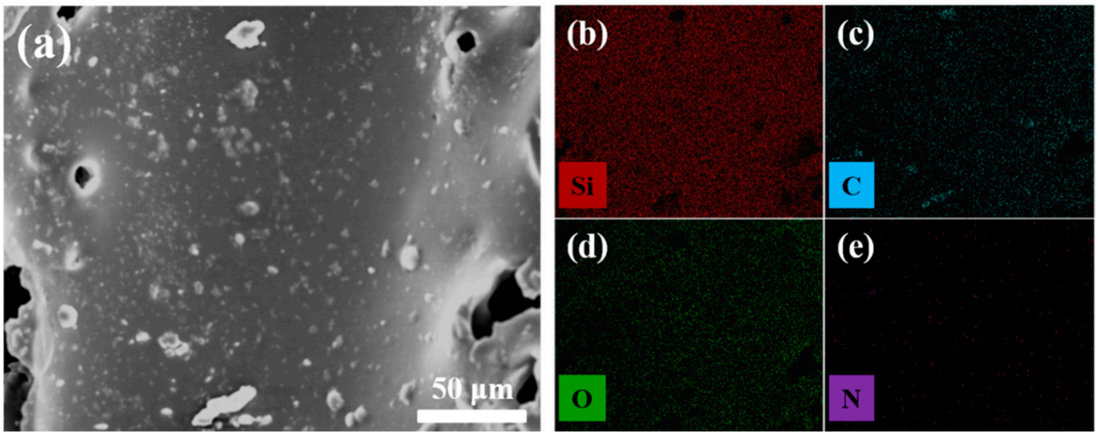

Figure S5. SEM image (**a**) of PDA/CNT-PDMS and corresponding EDS pictures (**b**–**e**).

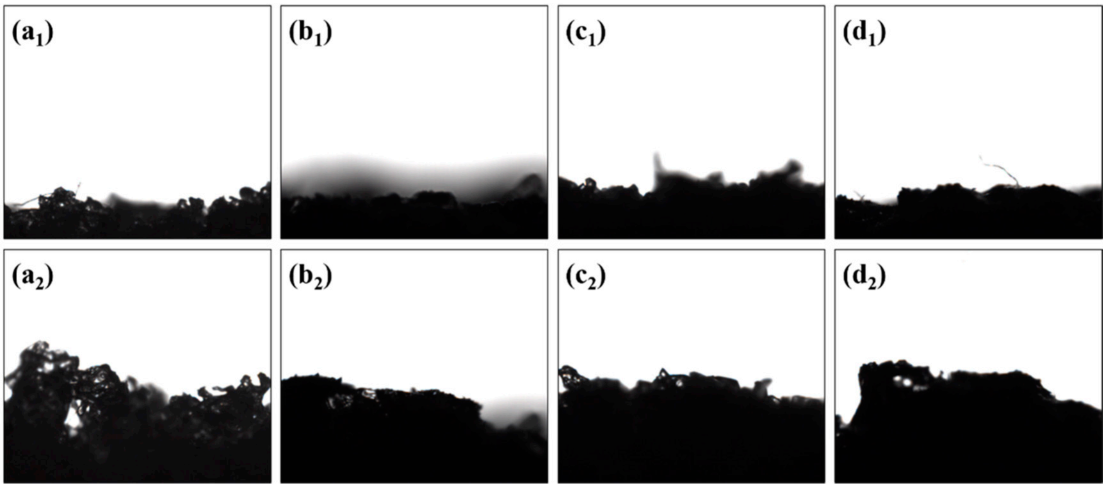

Figure 6. Optical images of a drop of cyclohexane deposited on PDMS (**a**1,**a**2), CNT-PDMS (**b**1,**b**2), PDA/PDMS (**c**1,**c**2), and PDA/CNT-PDMS (**d**1,**d**2) sponges immediately (**a**1–**d**1) and after a while (**a**2–**d**2).

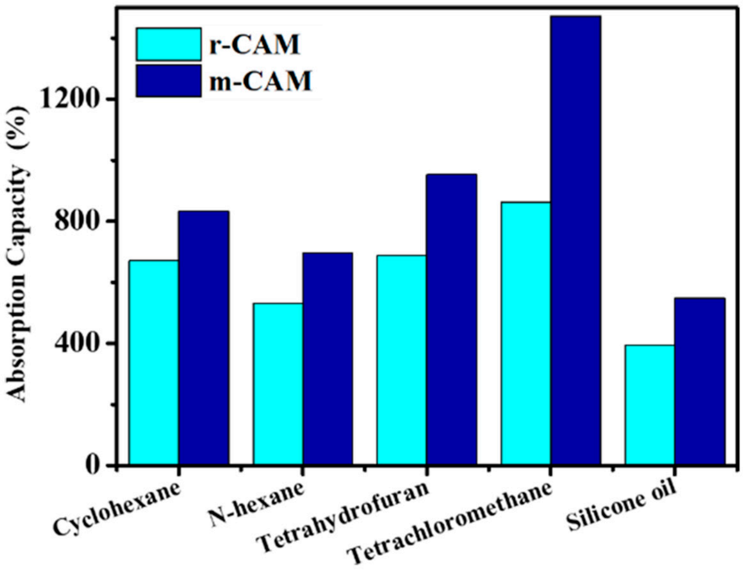

Figure S7. The influence of particle size of CAM on the absorption capacity of pure PDMS sponge for various oils.

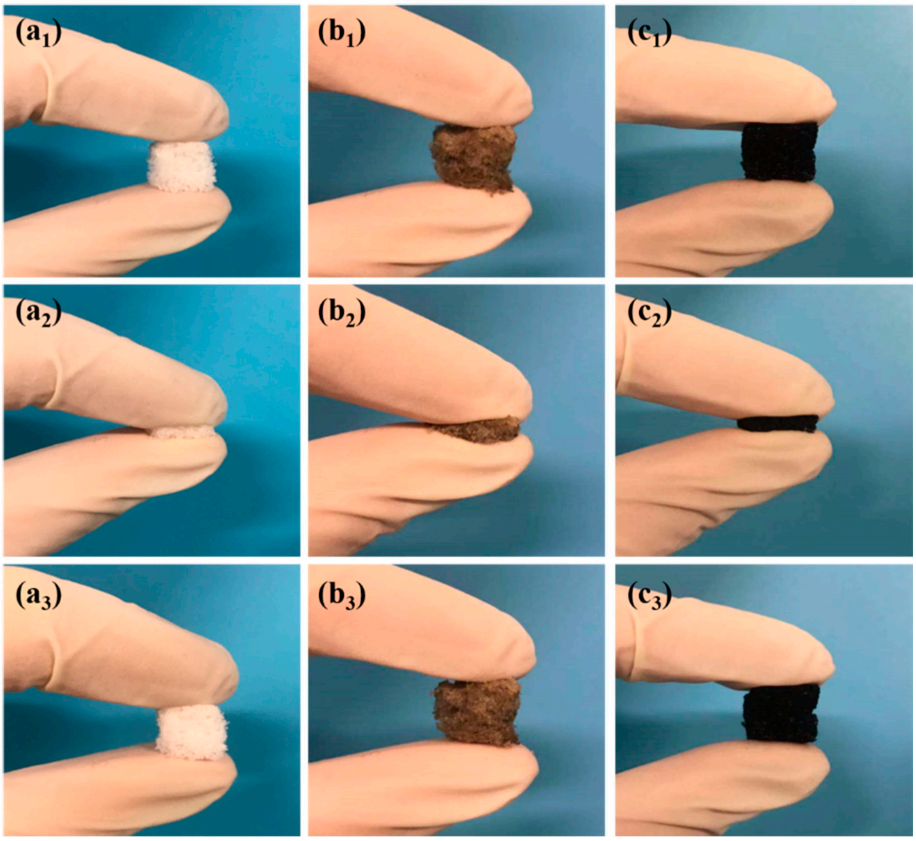

Figure S8. The elastic recovery of PDMS (**a**1–**a**3), PDA/CNT (**b**1–**b**3), and PDA/CNT-PDMS (**c**1–**c**3) sponges by compressing the initial sponge (**a**1–**c**1) to over 70% with an external force (**a**2–**c**2), and completely restored to its original shape after release (**a**3–**c**3).

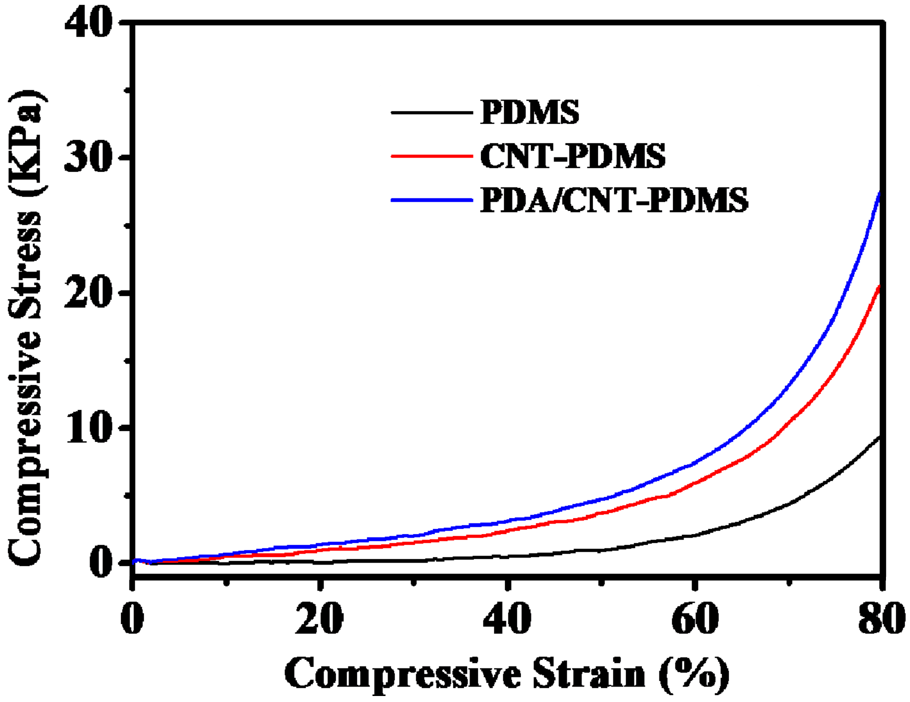

Figure S9. Compressive stress-strain curves of PDMS, CNT-PDMS, and PDA/CNT-PDMS sponges at the maximum strain of 80%.

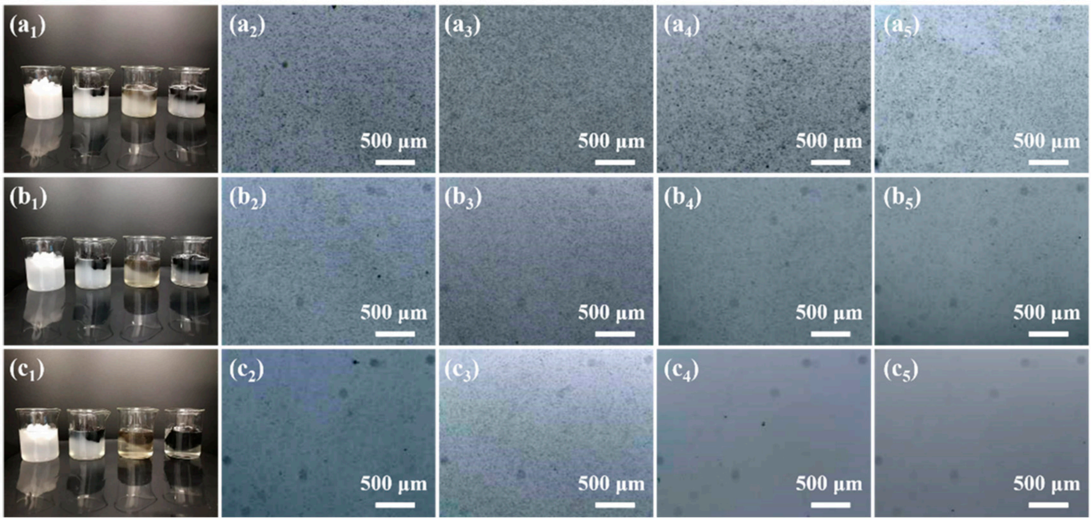

Figure S10. Photographs (**a**1–**c**1) of PDMS, CNT-PDMS, PDA/PDMS and PDA/CNT-PDMS sponges (from left to right) in toluene/water emulsion for 10 min (**a**1), 30 min (**b**1) and 50 min (**c**1) and the corresponding optical images (**a**2–**a**5, **b**2–**b**5, **c**2–**c**5) of emulsions.
